# Adherence to dietary recommendations for Swedish adults across categories of greenhouse gas emissions from food

**DOI:** 10.1017/S1368980017002300

**Published:** 2017-09-07

**Authors:** Camilla Sjörs, Fredrik Hedenus, Arvid Sjölander, Annika Tillander, Katarina Bälter

**Affiliations:** 1 Department of Medical Epidemiology and Biostatistics (MEB), Karolinska Institutet, Nobels väg 12a, SE-171 77 Stockholm, Sweden; 2 Division of Physical Resource Theory, Chalmers University of Technology, Gothenburg, Sweden; 3 Stanford Prevention Research Center, Stanford University School of Medicine, Stanford, CA, USA; 4 Division of Public Health Sciences, School of Health, Care and Social Welfare, Mälardalen University, Västerås, Sweden

**Keywords:** Climate change, Greenhouse gas emissions, Nutrient intake, Dietary recommendations, Adherence

## Abstract

**Objective:**

To explore associations between diet-related greenhouse gas emissions (GHGE), nutrient intakes and adherence to the Nordic Nutrition Recommendations among Swedish adults.

**Design:**

Diet was assessed by 4d food records in the Swedish National Dietary Survey. GHGE was estimated by linking all foods to carbon dioxide equivalents, using data from life cycle assessment studies. Participants were categorized into quartiles of energy-adjusted GHGE and differences between GHGE groups regarding nutrient intakes and adherence to nutrient recommendations were explored.

**Setting:**

Sweden.

**Subjects:**

Women (*n* 840) and men (*n* 627) aged 18–80 years.

**Results:**

Differences in nutrient intakes and adherence to nutrient recommendations between GHGE groups were generally small. The dietary intake of participants with the lowest emissions was more in line with recommendations regarding protein, carbohydrates, dietary fibre and vitamin D, but further from recommendations regarding added sugar, compared with the highest GHGE group. The overall adherence to recommendations was found to be better among participants with lower emissions compared with higher emissions. Among women, 27 % in the lowest GHGE group adhered to at least twenty-three recommendations compared with only 12 % in the highest emission group. For men, the corresponding figures were 17 and 10 %, respectively.

**Conclusions:**

The study compared nutrient intakes as well as adherence to dietary recommendations for diets with different levels of GHGE from a national dietary survey. We found that participants with low-emission diets, despite higher intake of added sugar, adhered to a larger number of dietary recommendations than those with high emissions.

Food and climate change are intimately related. The production of food generates greenhouse gas emissions (GHGE) and the increase in temperature due to global warming changes the ability to produce food^(^ ^1^ ^)^. The main diet-related greenhouse gases are methane, nitrous oxide and carbon dioxide. Methane is emitted from ruminants, rice cultivation and manure management; nitrous oxide from fertilizers applied to agricultural fields and manure management; and carbon dioxide from machinery, transport and food processing using fossil fuels^(^ ^2^ ^)^.

A Swedish environmental analysis group assigned by the Government, ‘The Green transition and competitiveness’, has investigated the implications of the target to limit global warming to 2°C^(^ ^3^ ^,^ ^4^ ^)^. If the amount of GHGE would be distributed equally among all individuals on Earth, the group concludes that annual GHGE needs to be reduced to between 1 and 2 t carbon dioxide equivalents (CO_2_e)/capita until 2050, to reach the climate goal. In comparison, from the consumption perspective, the total annual emissions per person in Sweden is close to 11 t CO_2_e^(^ ^3^ ^)^ and the annual emissions from food consumption is about 1·8 t CO_2_e^(^ ^5^ ^)^.

About three-quarters of the GHGE from Swedish food consumption originate from animal-based foods^(^ ^5^ ^)^. Generally, food products from ruminants have large carbon footprints (i.e. high emissions of GHGE), while plant-based foods have small carbon footprints^(^ ^2^ ^)^. Nevertheless, beef and dairy products are rich in certain nutrients, such as protein, SFA, Fe, Zn, vitamin B_12,_ Ca and vitamin D^(^ ^6^ ^,^ ^7^ ^)^. Therefore, reduced consumption of food products from ruminants may be more or less beneficial from a nutritional point of view, depending on how they are replaced. Legumes (beans, peas, lentils), whole grains, nuts and seeds, as well as fortified plant-based alternatives to dairy, are examples of sources of protein and micronutrients with small carbon footprints^(^ ^6^ ^)^.

Few studies have examined the carbon footprint and intakes of nutrients from self-selected diets^(^ ^8^ ^–^ ^11^ ^)^. Temme *et al*.^(^ ^8^ ^)^ and Scarborough *et al*.^(^ ^9^ ^)^ found that people with low diet-related GHGE had lower intakes of total fat, SFA and protein, as well as higher intakes of carbohydrates and dietary fibre, compared with people with high GHGE. They reported that the intakes of mono- and disaccharides^(^ ^8^ ^)^ and total sugars^(^ ^9^ ^)^ were higher in the group with low compared with high GHGE from food. Vieux *et al*.^(^ ^10^ ^)^ found that those with the lowest emissions had a higher consumption of free sugars, defined as added sugars and sugars naturally present in honey, syrups and fruit juices. However, none of these authors distinguished between sugars found naturally in food items and added sugar. Vieux *et al*.^(^ ^10^ ^)^ compared intakes of micronutrients between groups with varying levels of GHGE, however results were presented for indicators of overall nutritional quality and not for individual nutrients. We recently assessed intakes of selected nutrients as well as diet-related GHGE among Swedish adults using an FFQ, and found that intakes of some micronutrients were lower among participants who had low GHGE compared with those with high GHGE; however, differences were small^(^ ^11^ ^)^.

The aim of the present study was to compare intakes and adherence of all nutrients to the Nordic Nutrition Recommendations (NNR)^(^ ^6^ ^)^ between groups with low and high GHGE from food based on self-selected diets. We used data from a Swedish sample of 1467 women and men who filled out a 4 d food record, which was linked to life cycle assessment (LCA) data for food products.

## Participants and methods

Riksmaten adults 2010–11 is the most recent population-based national survey investigating food habits among adults in Sweden^(^ ^12^ ^)^. Data were collected during the period of May 2010 to July 2011 by Statistics Sweden on behalf of the National Food Agency (NFA). The study comprised a food record and a questionnaire, both of which were web-based.

An invitation including brief information about the study was sent out to a representative sample (*n* 5003) of women and men aged 18–80 years. A few days after the invitation letter was sent out, all subjects were contacted by an interviewer via telephone and given further instructions. Individuals who agreed to participate received additional written information, including personal logins to the web-based food record and the questionnaire, as well as pictures to aid the reporting of portion sizes. This information was reviewed by the participant and study personnel through a second telephone call before the first day of the food record. Participants were also given the option to perform the food record by telephone, and participants who chose this option received a printed version of the questionnaire. Height, weight and physical activity at work and during leisure time were self-reported through the questionnaire. The present study uses data primarily from the food records, which had a participation rate of 36 % (*n* 1797). The participation rate was higher among women (41 %) compared with men (31 %). Using the personal identity number of each participant, Statistics Sweden retrieved sex, age, income, education and country of birth from the Swedish Total Population Register.

### Dietary assessment

Diet was assessed using a 4 d self-assisted web-based food record, developed by the Swedish NFA, described in detail elsewhere^(^ ^12^ ^,^ ^13^ ^)^. The start date was randomly assigned to ensure that all seven days of the week were equally distributed. The reported distribution over the week was very consistent with the expected distributions of starting days. Expected distributions were 62·5 % weekdays, 12·5 % Fridays, 12·5 % Saturdays and 12·5 % Sundays. The reported distributions were 62·9 % weekdays, 12·8 % Fridays, 12·3 % Saturdays and 12·0 % Sundays. The participants were asked to report everything they ate and drank during four consecutive days and they could choose between more than 1900 food items and dishes. Portion sizes were estimated using pictures, household measures, number of portion units (cups, pieces, slices) or grams. After completion, the food records were linked to the Swedish food composition database^(^ ^14^ ^)^, which contains data on more than fifty nutrients. The database is managed by the Swedish NFA. Nutritional values of the food items and dishes in the food composition database are either analysed at certified laboratories on behalf of the NFA or calculated by the NFA^(^ ^15^ ^)^. For dishes with calculated nutritional values (‘calculated dishes’), the database includes information on recipes, with all ingredients and proportions listed.

### Diet-related greenhouse gas emissions

The combined climate effect of all greenhouse gases is expressed as kg CO_2_e/kg food product^(^ ^16^ ^)^. We collected GHGE data from LCA studies, aiming at representing the food consumption in Sweden, for about 100 food products/groups. The food records from the participants consisted of more than 100 food items. However, using just one specific LCA study to represent a specific food item may be misleading, as it may not represent the general production practices. Therefore, an average value from several LCA studies was used for each food product/group. For LCA data, see Table S1 in the online supplementary material. Different greenhouse gases have different effects on the Earth’s warming and the global warming potential (GWP) was developed to enable comparisons of the warming effect of different gases. Consistently, we compared different greenhouse gases using GWP with a 100-year time horizon. Based on the latest Intergovernmental Panel on Climate Change report, we used GWP=34 for methane, GWP=296 for nitrous oxide and GWP=1 for carbon dioxide to calculate CO_2_e^(^ ^17^ ^)^.

All LCA studies included GHGE from agriculture and its inputs, and the majority also included emissions up to and including the retail phase. We adjusted all LCA data to include the same system boundaries; for example, added standard emission factors from post-farm processes, including processing, packaging, distribution and retail^(^ ^18^ ^)^. Emissions after the retail phase (transport to households, storing and cooking, as well as from waste management) or emissions related to land-use change were not included.

Since participants could record food items in either raw or prepared form, LCA data were recalculated to the prepared form where appropriate. Both hydration (i.e. cooking of rice) and dehydration (i.e. cooking of meat) were adjusted for^(^ ^14^ ^)^. We further adjusted for unavoidable food losses (i.e. shell and bone)^(^ ^14^ ^)^, as well as avoidable food waste, both before and after food preparation^(^ ^19^ ^,^ ^20^ ^)^.

LCA data of GHGE per kg of food product/group were then linked to all food items and dishes registered in the food records. To estimate the GHGE of mixed dishes (e.g. meat pie or falafel with pita bread and salad), we used different approaches. For dishes with available information of specific recipes (i.e. ‘calculated dishes’), we linked each ingredient directly to LCA data. For mixed dishes without a specific recipe associated, we used common recipes and own estimations to determine a recipe. To save time and to simplify we decided that the recipes could include a maximum of three ingredients. However, the ingredients could be food products/groups or ‘calculated dishes’. For example, for falafel with pita bread and salad, we used the food products/groups ‘bread’ and ‘iceberg lettuce’ and the calculated dish ‘deep-fried falafel’ to estimate the GHGE of the dish.

We calculated GHGE separately for each recorded food item and dish for each participant, taking portion sizes into account. Finally, GHGE for each participant were summarized and an average daily kg CO_2_e was calculated.

### Reference values for assessing nutrient intakes

NNR 2012^(^ ^6^ ^)^ provides different reference values for assessing nutrient adequacy. We first describe reference values for energy-providing nutrients (macronutrients) and then reference values for vitamins and minerals (micronutrients).

The assessment of macronutrient intakes mainly concerns the energy distribution (as energy percentage, E%) from protein, fat and carbohydrates. To assess adequate intakes of protein, total fat, MUFA, PUFA and carbohydrates, the proportion of participants who have energy contributions within the recommended intake range was estimated. Furthermore, for macronutrients with a recommended upper threshold (SFA and added sugars), the proportion of the participants who are below this threshold was estimated. Likewise, for macronutrients with a recommended lower threshold (linoleic acid, α-linolenic acid and dietary fibre), the proportion that exceeds this level was estimated. Table S2 in the online supplementary material gives a detailed description of the reference values for assessing nutrient adequacy of macronutrients.

The estimated average requirement (AR) is the value primarily used to assess the risk for inadequate intakes of micronutrients in dietary surveys. The AR for Fe is higher for women in reproductive age than for postmenopausal women, and this was taken into account for the age intervals of 18–50 years and 51–80 years. Table S3 in the online supplementary material gives a detailed description of the reference values for assessing nutrient adequacy of micronutrients. However, AR is missing for a few micronutrients. Therefore, a lower intake level (LI) was used for K and a recommended intake (RI) was used for Mg and Na^(^ ^6^ ^)^. For comparison, RI values for all micronutrients can be found in Table S4 in the online supplementary material. These are reference values for nutrient intakes intended for dietary planning. In addition, Fig. S1 in the online supplementary material shows a visualization of the difference between AR and RI.

Adherences to twenty-seven nutrient recommendations were estimated taking account of the sex and age of the participants. The overall adherence to NNR was estimated by assessing the total number of recommendations that each participant fulfilled, i.e. between one and twenty-seven nutrient recommendations.

In Riksmaten adults 2010–11, intake of added refined sugar in the diet was calculated^(^ ^12^ ^)^. Each food/drink containing sucrose and monosaccharides was reviewed and the amount of naturally found and added sugar was estimated. To calculate added sugar (grams per day), the amounts of sucrose and monosaccharides from natural sources were subtracted from the total amounts of sucrose and monosaccharides in the diets. In addition, the E% from added sugar was calculated, referring to the proportion of the energy in the diet that the added sugar contributed.

### Statistical analyses

The Goldberg cut-off method was used to identify energy misreporters^(^ ^21^ ^)^. The cut-off value was calculated using the energy intake from the food record together with the obtained physical activity level (PAL) from the questionnaire.

Descriptive results are presented as medians and interquartile ranges (IQR; 25th–75th percentile) or percentages (%). GHGE was adjusted for total energy intake using the residual method^(^ ^22^ ^)^, by linear regression with total energy intake as independent variable and diet-related GHGE as dependent variable. The GHGE residuals provide a measure of the emission uncorrelated with total energy intake. However, since residuals have a mean of zero and include negative values they do not provide an intuitive sense of actual emissions, therefore a constant (the CO_2_e at the mean energy intake) was added to the residuals. Quartiles were used to split energy-adjusted CO_2_e into four groups. Medians and IQR of daily absolute nutrient intakes categorized by GHGE groups were calculated. Differences between GHGE groups were tested using the Kruskal–Wallis test. To test differences in proportions of the GHGE groups adhering to different nutrient recommendations, the *χ* ^2^ test was used.

The significance level was set to *α*=0·05. Analyses were performed using the statistical software package Stata version 14.0 and figures using R software version 3.3.0.

## Results

### Variation in diet-related greenhouse gas emissions

Of the 1797 participants completing the food records, 330 participants were excluded due to energy misreporting (165 women and 165 men). Characteristics of the 1467 participants included in the present study are shown in Table 1. The annual median crude diet-related GHGE was 1·5 t CO_2_e for women (ranging from 0·2 to 4·3 t) and 2·0 t CO_2_e for men (ranging from 0·7 to 6·1 t). Women had 26 % lower median crude GHGE than men. The daily absolute energy intake was 23 % lower among women compared with men. When we adjusted GHGE for the total energy intake, the difference between genders decreased. The annual median energy-adjusted GHGE was 1·7 t CO_2_e for women (ranging from 0·5 to 3·4 t) and 1·8 t CO_2_e for men (ranging from 0·3 to 4·5 t). Women had a 6 % lower median energy-adjusted diet-related emission than men. We categorized the participants into quartiles of energy-adjusted GHGE. The median emission in the lowest compared with the highest energy-adjusted GHGE groups was 1·27 and 2·26 t CO_2_e/year, respectively, for women (see Table 2(a)) and 1·25 and 2·62 t CO_2_e/year, respectively, for men (see Table 2(b)).

**Table 1:** Characteristics of the participants (*n* 1467) in the Riksmaten adults 2010–11 survey, Sweden

	Women (*n* 840)		Men (*n* 627)	
Characteristic	Median	IQR	Median	IQR
GHGE (kg CO_2_e/d)	4·1	1·8	5·5	2·5
GHGE (t CO_2_e/year)	1·5	0·7	2·0	0·9
Age (years)	48	29	50	25
PAL	1·7	0·2	1·7	0·2
Energy and selected nutrients				
Energy (kJ/d)	7740	2218	9990	3040
Protein (E%)*	17	4	17	4
Protein (g/d)	74	22	94	30
Carbohydrates (E%)*	46	8	45	8
Carbohydrates (g/d)	198	69	252	89
Dietary fibre (g/1000 kJ)	2·5	1·0	2·2	0·9
Dietary fibre (g/d)	19	8	22	9
Whole grains (g/d)	37	34	44	48
Added sugar (E%)*	10	6	10	6
Added sugar (g/d)	42	32	55	41
Vitamin D (µg/d)	5·8	4·7	6·6	5·7
Folate (µg/d)	249	100	276	103
Fe (mg/d)	10	4	12	4
Income (thousand Swedish kronor/year)				
Individual income	230	155	297	199
	%		%	
Highest education				
Compulsory school	10		14	
Upper secondary school	40		43	
<3 years after upper secondary school	17		16	
≥3 years after upper secondary school	34		27	
BMI category				
Underweight (<18·5 kg/m^2^)	3		0	
Normal weight (18·5–24·9)	61		51	
Overweight (25·0–29·9 kg/m^2^)	26		41	
Obese (≥30·0 kg/m^2^)	11		9	
Born in Sweden	90		92	

GHGE, greenhouse gas emissions; CO_2_e, carbon dioxide equivalents; PAL, physical activity level.

* E%, energy percentage (excluding energy from alcohol).

**Table 2(a):** Median and interquartile range (IQR) of daily absolute nutrient intakes by quartiles of increasing levels of dietary greenhouse gas emissions (GHGE), adjusted for total energy intake, among 840 women in the Riksmaten adults 2010–11 survey, Sweden

	GHGE (t dietary CO_2_e/year)								
	Quartile 1		Quartile 2		Quartile 3		Quartile 4		
	0·55–1·45 (median 1·27)		1·45–1·71 (median 1·58)		1·71–2·03 (median 1·84)		2·03–3·37 (median 2·26)		
	Median	IQR	Median	IQR	Median	IQR	Median	IQR	*P* value*
Energy and macronutrients									
Energy (kJ/d)	8506	2173	7551	2098	7330	2031	7616	2220	0·0001
Protein (E%)†	15	3	17	3	17	4	18	3	0·0001
Protein (g/d)	73	22	72	22	71	20	78	22	0·0002
Carbohydrates (E%)†	47	9	46	8	46	7	44	8	0·0001
Carbohydrates (g/d)	227	70	196	61	190	63	188	59	0·0001
Dietary fibre (g/1000 kJ)	2·6	1·1	2·6	1·0	2·4	0·9	2·4	0·9	0·0007
Dietary fibre (g/d)	22	10	19	8	18	7	18	8	0·0001
Whole grains (g/d)	43	45	38	31	35	36	33	28	0·0003
Added sugar (E%)†	11	7	9	5	11	6	9	6	0·0003
Added sugar (g/d)	52	43	39	25	43	28	39	31	0·0001
Total fat (E%)†	35	7	35	9	35	6	36	7	0·3
Total fat (g/d)	79	30	69	28	68	28	71	28	0·0001
SFA (E%)†	13	4	13	4	13	3	14	4	0·3
SFA (g/d)	30	13	27	12	26	12	27	13	0·002
Cholesterol (mg/d)	245	147	244	155	253	111	267	145	0·04
MUFA (E%)†	13	3	13	4	13	3	13	3	0·2
MUFA (g/d)	29	11	25	11	26	10	27	11	0·0001
PUFA (E%)†	6·1	3·0	5·3	2·0	5·2	1·9	5·4	2·1	0·0001
PUFA (g/d)	14	8	11	5	10	5	11	5	0·0001
*n*-3 FA (E%)†,‡	1·3	0·7	1·1	0·6	1·2	0·5	1·2	0·6	0·003
*n*-3 FA (g/d)‡	2·9	1·7	2·2	1·4	2·3	1·5	2·4	1·1	0·0001
Linoleic acid (E%)†	4·4	2·3	3·8	1·7	3·7	1·4	3·9	1·8	0·0001
Linoleic acid (g/d)	10·4	6·2	7·7	4·1	7·3	3·6	7·8	4·3	0·0001
α-Linolenic acid (E%)†	1·0	0·5	0·9	0·5	0·9	0·4	0·9	0·4	0·02
α-Linolenic acid (g/d)	2·2	1·3	1·8	1·0	1·8	1·0	1·9	1·0	0·0001
Vitamins									
Retinol (µg/d)	486	304	462	286	416	266	426	273	0·006
β-Carotene (µg/d)	1797	2272	1811	2330	1766	1922	1799	2121	0·5
Vitamin A (RE/d)§	757	451	715	466	644	329	666	430	0·0004
Vitamin D (µg/d)	6·7	5·7	5·6	4·2	5·6	5·1	5·5	4·2	0·004
Vitamin E (α-TE/d)║	12	5	10	5	10	4	10	5	0·0001
Thiamin (mg/d)	1·2	0·5	1·1	0·4	1·1	0·3	1·1	0·4	0·002
Riboflavin (mg/d)	1·5	0·6	1·4	0·6	1·4	0·5	1·4	0·5	0·3
Niacin (NE/d)¶	32	10	31	11	31	9	34	10	0·002
Vitamin B_6_ (mg/d)	1·8	0·7	1·7	0·7	1·8	0·7	1·9	0·8	0·053
Folate (µg/d)	276	113	248	106	238	91	242	85	0·0001
Vitamin B_12_ (µg/d)	4·5	3·6	4·4	3·0	4·5	2·9	4·9	2·9	0·06
Vitamin C (mg/d)	96	73	90	68	89	77	99	69	0·4
Minerals									
Ca (mg/d)	908	375	846	378	829	318	824	318	0·02
P (mg/d)	1341	382	1249	422	1203	364	1279	416	0·0006
K (mg/d)	3082	889	2882	926	2923	884	3020	910	0·07
Mg (mg/d)	348	113	308	105	289	97	307	90	0·0001
Fe (mg/d)	10	4	9	3	9	4	10	3	0·0001
Zn (mg/d)	9	3	9	3	10	3	11	3	0·0001
Se (µg/d)	42	20	39	19	40	20	44	21	0·02
Na (mg/d)	2834	978	2745	1085	2718	931	2932	805	0·01

CO_2_e, carbon dioxide equivalents; FA, fatty acids.

* Kruskal–Wallis test.

† E%, energy percentage (excluding energy from alcohol).

‡ To calculate *n*-3 FA in the present study, α-linolenic acid (18:3*n*-3), EPA (20:5*n*-3) and DHA (22:6*n*-3) were summarized.

§ RE, retinol equivalent; 1 RE=1 μg retinol=12 μg β-carotene.

║ α-TE, α-tocopherol equivalents.

¶ NE, niacin equivalents.

**Table 2(b):** Median and interquartile range (IQR) of daily absolute nutrient intakes by quartiles of increasing levels of dietary greenhouse gas emissions (GHGE), adjusted for total energy intake, among 627 men in the Riksmaten adults 2010–11 survey, Sweden

	GHGE (t dietary CO_2_e/year)								
	Quartile 1		Quartile 2		Quartile 3		Quartile 4		
	0·35–1·51 (median 1·25)		1·51–1·81 (median 1·66)		1·81–2·24 (median 2·02)		2·25–4·50 (median 2·62)		
	Median	IQR	Median	IQR	Median	IQR	Median	IQR	*P* value*
Energy and macronutrients									
Energy (kJ/d)	10494	2664	9776	2828	9432	2998	10 189	3345	0·0004
Protein (E%)†	16	3	16	3	17	3	19	4	0·0001
Protein (g/d)	91	29	91	26	90	27	106	33	0·0001
Carbohydrates (E%)†	47	9	46	8	45	8	43	8	0·0001
Carbohydrates (g/d)	288	94	252	82	241	75	242	87	0·0001
Dietary fibre (g/1000 kJ)	2·4	0·9	2·3	0·9	2·1	0·9	2·0	0·8	0·0001
Dietary fibre (g/d)	25	12	22	7	20	9	21	9	0·0001
Whole grains (g/d)	52	54	45	46	39	46	41	46	0·002
Added sugar (E%)†	11	6	10	6	10	6	9	6	0·0001
Added sugar (g/d)	62	48	55	39	53	36	49	36	0·0001
Total fat (E%)†	35	8	35	8	37	8	36	8	0·1
Total fat (g/d)	96	39	91	33	89	37	94	38	0·07
SFA (E%)†	13	5	14	4	14	4	14	4	0·2
SFA (g/d)	36	19	34	15	34	16	36	13	0·09
Cholesterol (mg/d)	302	167	298	129	315	145	354	192	0·0001
MUFA (E%)†	13	3	13	3	13	3	13	2	0·047
MUFA (g/d)	35	16	34	12	33	14	35	14	0·2
PUFA (E%)†	5·4	2·3	5·2	1·9	5·4	2·4	5·2	2·3	0·6
PUFA (g/d)	15	8	13	6	14	8	14	7	0·02
*n*-3 FA (E%)†,‡	1·2	0·6	1·1	0·7	1·1	0·6	1·1	0·5	0·1
*n*-3 FA (g/d) ‡	3·3	2·0	2·9	1·7	2·7	1·6	2·7	1·4	0·003
Linoleic acid (E%)†	4·0	1·7	3·7	1·4	3·9	1·9	3·8	1·8	0·3
Linoleic acid (g/d)	11·3	6·5	9·3	4·4	10·1	5·8	10·0	5·4	0·02
α-Linolenic acid (E%)†	0·9	0·4	0·9	0·4	0·9	0·4	0·9	0·4	0·5
α-Linolenic acid (g/d)	2·6	1·5	2·3	1·4	2·3	1·3	2·4	1·1	0·02
Vitamins									
Retinol (µg/d)	601	409	526	346	488	329	554	381	0·04
β-Carotene (µg/d)	1757	2080	1376	1798	1333	1831	1280	1782	0·2
Vitamin A (RE/d)§	855	491	729	446	745	383	750	428	0·002
Vitamin D (µg/d)	8·2	6·9	6·4	5·9	5·9	4·6	6·1	5·4	0·0006
Vitamin E (α-TE/d)║	13	6	12	4	12	6	12	6	0·002
Thiamin (mg/d)	1·5	0·5	1·4	0·5	1·3	0·4	1·5	0·5	0·0009
Riboflavin (mg/d)	1·8	0·8	1·7	0·6	1·6	0·6	1·8	0·7	0·0002
Niacin (NE/d)¶	40	13	41	12	39	13	46	16	0·0001
Vitamin B_6_ (mg/d)	2·3	1·0	2·2	0·7	2·2	0·9	2·4	0·9	0·02
Folate (µg/d)	300	112	275	69	261	97	270	116	0·0001
Vitamin B_12_ (µg/d)	6·1	4·0	5·2	3·9	5·5	3·1	5·8	3·9	0·008
Vitamin C (mg/d)	94	84	85	63	92	78	84	88	0·3
Minerals									
Ca (mg/d)	1087	493	912	405	913	372	981	478	0·0001
P (mg/d)	1656	535	1552	414	1494	434	1728	567	0·0001
K (mg/d)	3711	1171	3546	978	3329	1039	3713	1251	0·0004
Mg (mg/d)	408	131	381	98	351	129	387	142	0·0001
Fe (mg/d)	12	4	11	4	11	4	13	5	0·0001
Zn (mg/d)	12	4	12	4	12	4	15	4	0·0001
Se (µg/d)	52	24	49	21	49	23	53	27	0·008
Na (mg/d)	3565	1178	3524	977	3587	1282	4014	1246	0·0001

CO_2_e, carbon dioxide equivalents; FA, fatty acids.

* Kruskal–Wallis test.

† E%, energy percentage (excluding energy from alcohol).

‡ To calculate *n*-3 FA in the present study, α-linolenic acid (18 : 3*n*-3), EPA (20 : 5*n*-3) and DHA (22 : 6*n*-3) were summarized.

§ RE, retinol equivalent; 1 RE=1 μg retinol=12 μg β-carotene.

║ α-TE, α-tocopherol equivalents.

¶ NE, niacin equivalents.

### Age, BMI and physical activity level

Men in the lowest energy-adjusted GHGE group were older than men in the highest group (median 56 *v*. 47 years) but for women there were no differences regarding age (data not shown). Further, there were no differences regarding BMI and PAL between the lowest and highest energy-adjusted GHGE groups for either women or men (data not shown).

### Variation in nutrient intakes

Tables 2(a) and 2(b) show medians and IQR of daily absolute nutrient intakes by quartiles of increasing levels of energy-adjusted GHGE for women and men, respectively. Differences in nutrient intakes were small between the lowest and highest emission groups. The largest differences were seen for the intakes of protein and carbohydrates. The proportion of energy from protein intake was about 20 % higher in the highest GHGE groups compared with the lowest for both women and men. In contrast, the proportions of energy coming from carbohydrates, as well as from added sugar, were higher in the lowest compared with the highest GHGE groups. The proportion of energy from added sugar was 20 % higher for women in the lowest GHGE group compared with women in the highest group, and 30 % higher when comparing men in the two groups. Also, the lowest GHGE groups had higher intakes of dietary fibre and whole grains compared with the highest groups. The consumption of whole grains by the women and men in the lowest GHGE groups was 30 and 26 % higher, respectively, in comparison to the highest groups.

### Variation in adherence to the Nordic Nutrition Recommendations

**Fig. 1:**
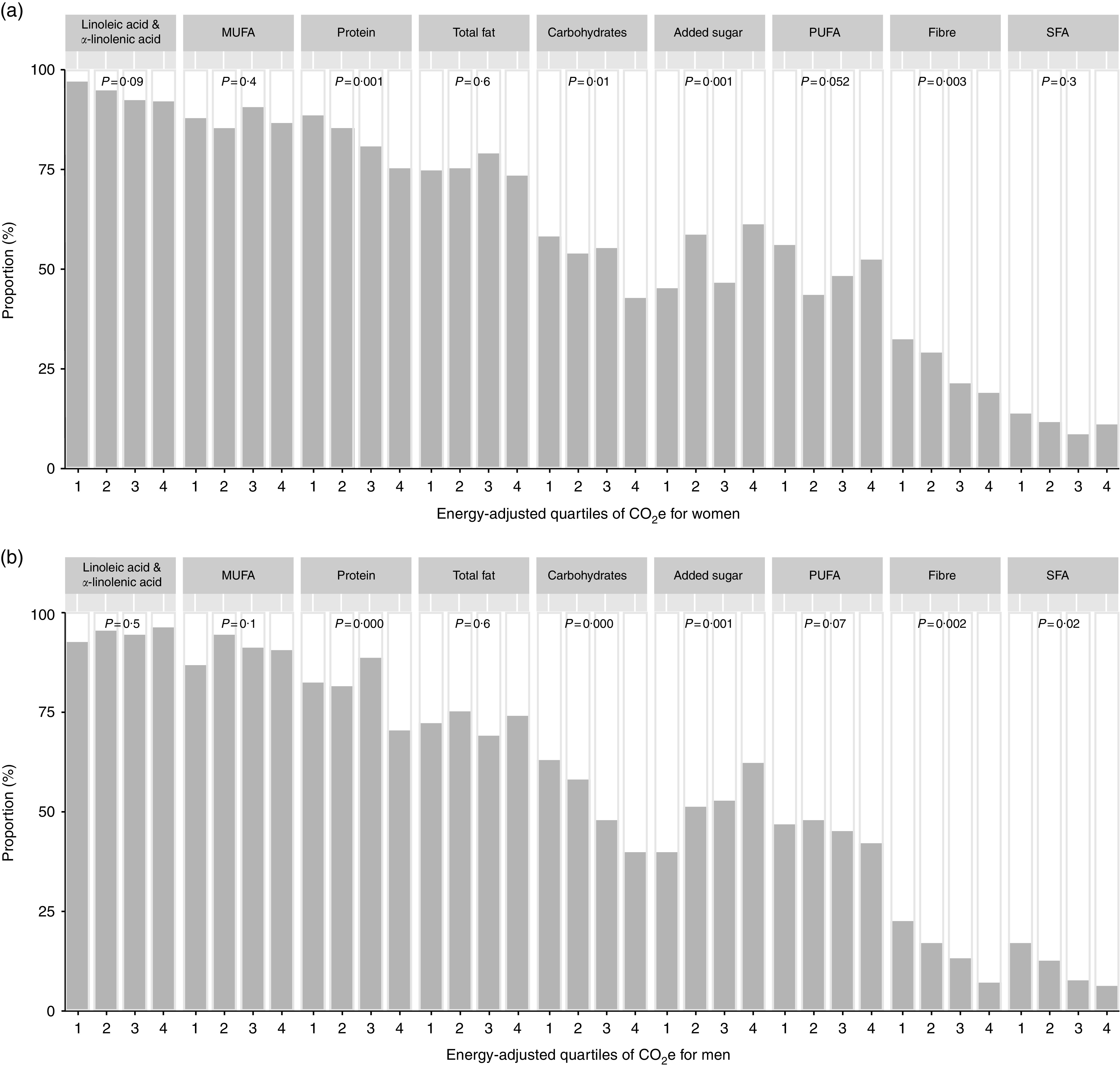
Proportion of participants adhering to the Nordic Nutrition Recommendations 2012 for macronutrients (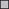, adhering; 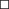, not adhering), by quartiles of increasing levels of dietary GHGE adjusted for total energy intake, among (a) 840 women and (b) 627 men, Riksmaten adults 2010–11 survey, Sweden. Quartile 1 is the lowest and 4 the highest GHGE group. *P* values are from *χ* ^2^ test (GHGE, greenhouse gas emissions; CO_2_e, carbon dioxide equivalents)

**Fig. 2:**
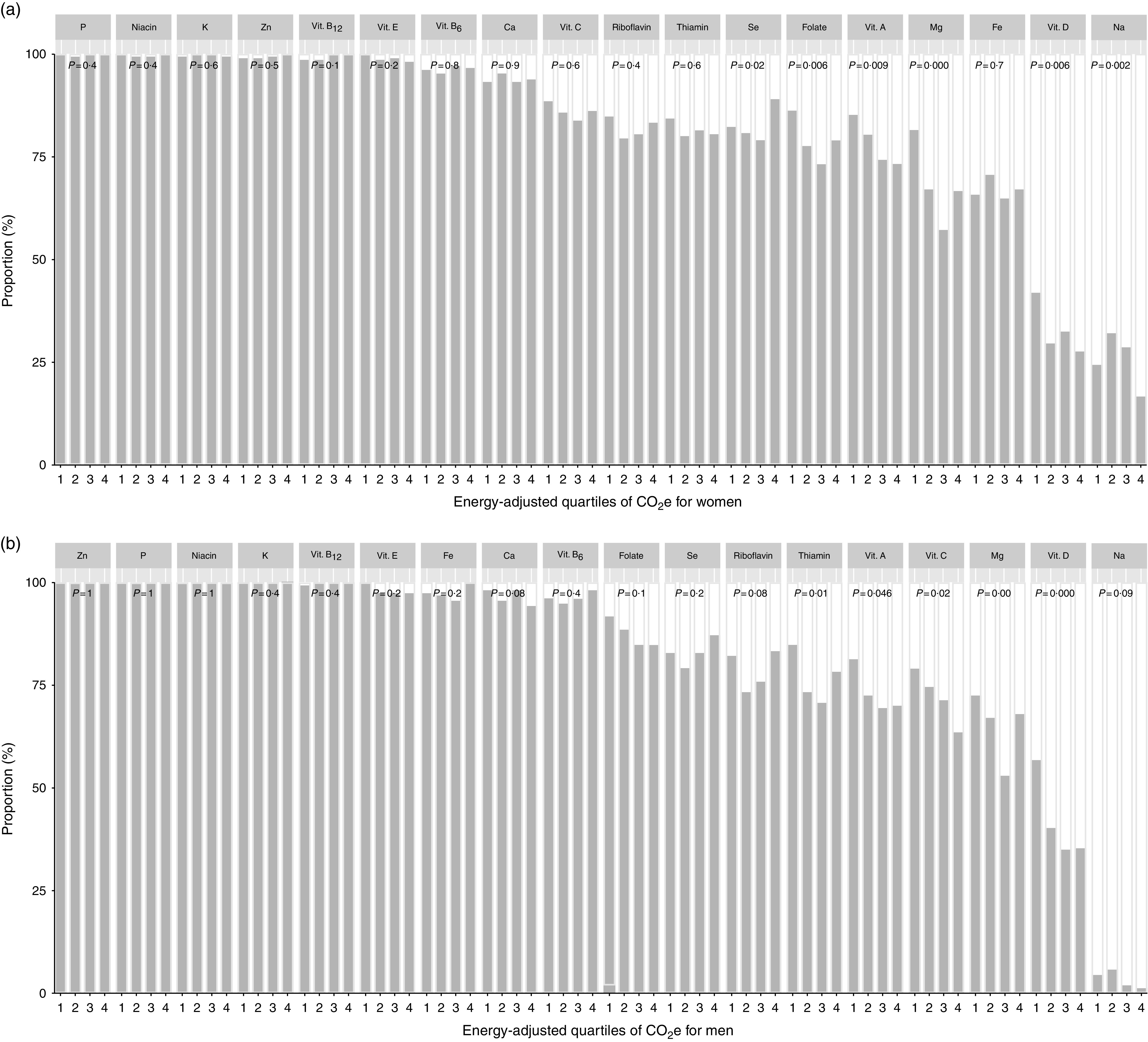
Proportion of participants adhering to the Nordic Nutrition Recommendations for micronutrients (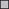, adhering; 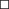, not adhering), by quartiles of increasing levels of dietary GHGE adjusted for total energy intake, among (a) 840 women and (b) 627 men, Riksmaten adults 2010–11 survey, Sweden. Quartile 1 is the lowest and 4 the highest GHGE group. *P* values are from *χ* ^2^ test (GHGE, greenhouse gas emissions; CO_2_e, carbon dioxide equivalents; Vit., vitamin)

Even though there were only small differences between the median intakes of nutrients between the GHGE groups, we analysed whether the proportion of individuals adhering to NNR differed between groups. Figures 1(a) and 1(b) show the proportion of women and men, respectively, who adhered to NNR for macronutrients (recommended intake ranges for protein, total fat, MUFA, PUFA and carbohydrates, and recommended thresholds for SFA, added sugars, linoleic acid, α-linolenic acid and dietary fibre) by quartiles of increasing levels of energy-adjusted GHGE. The proportions of women and men adhering to NNR for micronutrients (adherence to AR for fifteen nutrients, LI for one nutrient and RI for two nutrients) are shown in Figs 2(a) and 2(b). There were large differences between nutrients, but differences between GHGE groups within the same nutrient were generally small. In addition, for half of the macronutrients and two-thirds of the micronutrients, the differences between the GHGE groups were not statistically significant.

There were differences in the proportion of individuals adhering to the recommendation for protein among GHGE groups. In the lowest GHGE group, 88 % of the women and 82 % of the men adhered to protein recommendation, compared with 73 and 69 % for women and men, respectively, in the highest group. In the highest GHGE group, practically all of those not adhering to the recommendation consumed more protein than recommended. In contrast, more than half of those in the lowest GHGE group not adhering to the recommendation consumed less protein than recommended. The consumption of carbohydrates in the lowest GHGE groups was more in line with the recommendation, compared with the consumption in the highest GHGE groups. Moreover, 32 % of the women in the lowest GHGE group adhered to dietary fibre recommendation, while only 18 % did in the highest GHGE group. Among men, 22 % in the lowest group adhered to the dietary fibre recommendation and only 7 % in the highest group adhered. In contrast, the lowest GHGE groups adhered to the maximum recommended level of added sugar to a lesser extent than the highest groups. About 45 % of the women in the lowest group and 61 % in the highest group adhered to the added sugar recommendation. For men, the corresponding figures were 39 and 62 %.

Na and vitamin D were the micronutrients with lowest proportions of adherence. For Na, 24 % of the women in the lowest GHGE group adhered to NNR compared with 17 % in the highest group. The adherence to the Na recommendation was lower among men, but there was no difference between GHGE groups. In the lowest GHGE group, 41 % of the women and 57 % of the men adhered to the vitamin D recommendation, compared with 26 and 35 % in the highest groups, respectively. The adherence to Fe recommendation was lower among women than men, however there were no differences between GHGE groups. For comparison, the proportions of women and men adhering to RI for all eighteen micronutrients are shown in Figs S2(a) and S2(b), respectively, in the online supplementary material.

### Variation in total number of fulfilled recommendations

Table 3 shows proportions of women and men adhering to different total numbers of recommendations, by quartiles of increasing levels of energy-adjusted GHGE. Only a small proportion of the participants adhered to twenty-five recommendations or more. However, looking at the proportions adhering to twenty-three recommendations or more, we found differences between the GHGE groups. Among women, 27 % in the lowest GHGE group and 12 % in the highest emission group adhered to at least twenty-three recommendations. For men, the corresponding figures were 17 and 10 %. Figures S3(a) and S3(b) in the online supplementary material show boxplots of the total number of fulfilled nutrient recommendations by quartiles of increasing levels of energy-adjusted GHGE, for women and men, respectively. The median number of total recommendations adhered to was 21, 20, 20 and 20, respectively, for quartile 1 to 4, for both women and men.

**Table 3:** Proportions of women and men adhering to different total numbers of recommendations in the Nordic Nutrition Recommendations 2012, by quartiles of increasing levels of greenhouse gas emissions (GHGE) adjusted for total energy intake. Quartile 1 is the lowest and 4 the highest GHGE group

	Quartiles of GHGE				Quartiles of GHGE			
Total number of recommendations	1	2	3	4	1	2	3	4
adhered to	Women (%)				Men (%)			
6–12	0	1	0	1	0	1	1	0
13–16	6	10	14	13	8	13	16	15
17–18	13	16	20	14	11	12	18	17
19–20	26	24	24	28	25	27	27	31
21–22	28	32	26	30	38	31	28	27
23–24	22	14	14	12	17	13	10	9
25–26	4	2	1	0	0	3	0	1

## Discussion

The present study shows that differences in nutrient intakes between participants with the smallest and largest carbon footprints from diet are generally small. Also, differences in adherence to individual nutrient recommendations are small. However, when looking at the total adherence, we see that participants with the smallest carbon footprint from diet on average fulfil a larger number of recommendations than participants with the largest carbon footprint. Our finding that diets with the smallest carbon footprint are more in line with nutrient recommendations, compared with diets with the largest footprint, is in contrast to Vieux *et al*.^(^ ^10^ ^)^, who found higher nutritional quality among those with a large carbon footprint. In the study by Vieux *et al.* ^(^ ^10^ ^)^, participants with a healthier diet consumed more vegetables, fruits and fish and less sweets and salted snacks than participants with a less healthy diet. However, the consumption of beef, lamb and pork was the same for both groups.

Participants with the smallest carbon footprint have intakes of protein, carbohydrates and dietary fibre that are more in line with recommendations and this result is consistent with results from previous studies^(^ ^8^ ^,^ ^9^ ^,^ ^11^ ^)^. Moreover, we show that those with the smallest footprint consume most whole grains. In contrast, we also show that participants with the largest carbon footprint have an intake of added sugar that is more in line with recommendations. Our finding that the more climate-friendly diet includes more added sugar than the less climate-friendly diet agrees with other studies on the intakes of mono- and disaccharides^(^ ^8^ ^)^, total sugars^(^ ^9^ ^)^ and free sugars^(^ ^10^ ^)^. We have not studied which food items the added sugar comes from in the lowest and highest GHGE groups; however, among all participants in the Swedish National Dietary Survey, the main sources of added sugar were sweetened drinks, buns, cookies, cakes, sweets and chocolate^(^ ^7^ ^)^. Therefore, one needs to keep the concern expressed by Ridoutt *et al.* in mind, that a diet with small carbon footprint focusing only on reducing the intake of animal products may lead to excess consumption of energy-dense, nutrient-poor, non-core (or discretionary) foods^(^ ^23^ ^)^.

A diet with small carbon footprint is not necessarily more nutritious than a diet with large carbon footprint^(^ ^23^ ^,^ ^24^ ^)^. A systematic review of dietary scenarios^(^ ^24^ ^)^ reported that diets with low GHGE were often low in essential micronutrients. In the present study, there are only small differences regarding intakes of vitamins and minerals between participants with small and large carbon footprint. For vitamin D, intake is higher among the participants with the lowest emissions, although the intakes are still substantially below the AR. This is in contrast to our previous study with data from a validated FFQ^(^ ^11^ ^,^ ^25^ ^)^, where the intake of vitamin D was highest among participants with highest GHGE. The discrepancy between our studies may be explained by the different dietary assessment methods and study populations. The present study is more representative of Sweden, with a greater distribution of age and education levels, as well as wider geographical coverage, compared with our previous study. In addition, in the present study we use more detailed LCA data for seafood and dairy products, which are foods rich in vitamin D. Furthermore, GHGE for most seafood in the present study is lower than in our previous study. It is possible that participants with high seafood consumption end up in the lowest GHGE group in the present study, but not in our previous study. There were no differences in proportions adhering to Fe recommendations between low and high GHGE groups and this confirms the results from our previous study^(^ ^11^ ^)^. However, (haem) Fe in meat is generally more efficiently absorbed than (non-haem) Fe in grains and other plant-based foods^(^ ^6^ ^)^, and we did not study bioavailability of Fe in the different GHGE groups. Overall, the nutrients of concern in the diet of the Swedish population are high intakes of SFA, Na (from salt, NaCl) and added sugar, and low intakes of fibre, whole grains, vitamin D, folate and Fe (especially for women of reproductive age)^(^ ^7^ ^)^. In the present study, the lowest GHGE diet showed a positive picture for most critical nutrients. Therefore, although the attempt to look at dietary guidelines and food-related carbon footprint at the same time is complex, the results indicate that intakes of nutrients do not need to be a concern when people adopt to a more climate-friendly diet in a well-nourished population. However, the high intake of added sugar in the eating pattern with low GHGE is a concern.

Acknowledging that food preferences differ greatly between individuals, the numerous proposed climate-friendly scenarios, often with reduced meat intake, are more or less realistic for different consumers^(^ ^26^ ^,^ ^27^ ^)^. Through interviews with consumers Macdiarmid *et al.* learned that the opinion around eating meat is associated with important personal, social as well as cultural values^(^ ^26^ ^)^. The authors suggest that, since eating meat is the normalized dietary habit in most affluent countries, a shift in social norms is needed for consumers to eat less meat. In a recent review, Hartmann and Siegrist found that consumers have low willingness to change meat consumption behaviour and the authors highlighted the need for studies exploring motivational aspects of sustainable food choices^(^ ^27^ ^)^.

NNR are nutrient-based dietary guidelines, not food-based, and therefore we have focused on comparing the nutrient intakes between GHGE groups. As further steps towards increased knowledge on climate-friendly and healthy food habits, future studies comparing intakes of different food groups among those adhering to a larger number of nutrient recommendations are warranted. In the Swedish NFA’s report on the national survey, the contributions of nutrients from different food groups are presented for women and men together^(^ ^7^ ^)^. The main sources of vitamin D in the diet were fish, margarine, dairy products and meat, and the main sources of Fe were bread, meat and vegetables (including legumes).

There are several strengths and limitations of the present study. First, our analyses are based on food intake data from a national dietary survey using a comprehensive dietary assessment method. This range of different realistic food choices enables us to compare the nutrient intakes of participants with small and large carbon footprints from diet without having to create theoretical diets. An important strength of this observational study is that a representative sample was invited to participate. However, the participation rate in Riksmaten was low and therefore the results are not fully representative for the total Swedish population^(^ ^7^ ^)^. Women who declined to participate were evenly distributed among the different age groups, while non-participating men were more likely to be younger. Three out of four men aged 18–30 years declined to participate. Non-participation was also high among those born outside Sweden. The level of education differed between participants (14 % had compulsory school and 44 % had university/college degree as highest level) and non-participants (23 % compulsory school and 27 % university/college degree). Finally, income was somewhat higher among participants compared with non-participants. On average, participants in Riksmaten had higher level of education than the general population in Sweden. During 2010–11, 20 % of the women and 16 % of the men in Sweden had studied for three or more years after upper secondary school^(^ ^28^ ^)^, compared with 34 and 27 %, respectively, in Riksmaten. The proportion of overweight and obesity of the participants was slightly lower compared with the total adult population in Sweden. In 2010, 43 % of the women and 55 % of the men were overweight or obese^(^ ^29^ ^)^, compared with 37 and 50 %, respectively, in the present study.

The present study is the first to assess adequate intakes of all macronutrients and micronutrients compared with the NNR^(^ ^6^ ^)^, for diets with different levels of GHGE. We have made comparisons separately for women and men and have taken into account that recommendations differ between ages. The proportion of adherence to nutrition recommendations may be an important indicator of the public health effect of food habits with low and high GHGE. In our previous study, using a validated FFQ^(^ ^11^ ^,^ ^25^ ^)^, we compared the micronutrient intakes with RI, using goals for menu planning expressed as nutrients/MJ for ages 6–65 years. However, RI should preferably be used when planning diets for groups and the requirements are lower for almost all individuals. In the present study we use reference values for assessing nutrient intakes: AR. The proportion of participants who have a micronutrient intake below the AR have an increased risk of inadequate intake. However, we do not distinguish between participants who are just below the AR and participants who are far below AR. Moreover, we have not taken account of the use of dietary supplements.

Food records capture the whole diet, which is an advantage when comparing intakes with nutrient recommendations. To reduce the participation burden, pictures were used to estimate portion sizes. Nevertheless, keeping a food record may influence food choices and it is common that participants under-report their food intake, more or less consciously, and eat healthier than they usually would while recording their food intake. Temme *et al.* assessed diet with 24 h dietary recalls and found that more under-reporting occurred in the low- compared with the high-GHGE group^(^ ^8^ ^)^. However, they did not adjust the GHGE for total energy intake. Although we excluded participants who were under- and over-reporters based on the Goldberg cut-off method^(^ ^21^ ^)^, we do not know whether the included participants, consciously or unconsciously, have misreported their intake.

All food items and dishes in the food records were linked to LCA data aiming to be representative for Swedish consumption. However, LCA data are based on many assumptions. There is great variation in emissions for some foods (e.g. seafood) and a limitation in the present study is that we did not have any information on origin and capture methods for seafood reported in the food records, and therefore we had to make assumptions. A strength is that we have adjusted the LCA data to include the same system boundaries, taken changes in weight due to food preparation into account, as well as food waste both before and after food preparation. We had information about ingredients for about half of the mixed dishes and assumptions were made for the remaining dishes.

The energy-adjusted carbon footprint in the present study varied from 0·3 to 4·5 t CO_2_e/year, and annual median energy-adjusted emissions were 1·7 and 1·8 t CO_2_e for women and men, respectively. We adjusted the GHGE for total energy intake for two reasons. First, under-reporting of food intake is common in food surveys. Second, it would be misleading to compare diet-related GHGE between participants who eat small amounts of food and participants who eat large amounts of food due to variation in body size or energy expenditure. We are interested in the quality of the food (different food products) rather than the quantity of food intake (different portion sizes). The emission level in the current study is similar to the emissions in our previous study with Swedish dietary data assessed with a validated FFQ, where the energy-adjusted diet-related GHGE were 1·7 and 1·9 t CO_2_e/year for women and men, respectively^(^ ^11^ ^,^ ^25^ ^)^. The median carbon footprint from the current Swedish food habits is as large as the proposed total carbon footprint (1–2 t CO_2_e/capita per year) required to reach the global 2°C target^(^ ^3^ ^)^; however, there is a wide variety in emissions between different participants. We can therefore conclude that there is an urgent need to reduce the diet-related carbon footprint and a potential to do so with good nutritional status.

The present study may serve as a basis for policy making, decisions and actions to shift eating patterns on many levels, from the government, food industry, education system, health care, to consumer levels. A review of the evidence of the effectiveness of interventions aimed at shifting diets to more sustainable and healthy directions by Garnett *et al.* studied several different approaches for interventions^(^ ^30^ ^)^. These approaches included fiscal measures (including taxes), changing the context, defaults and norms of consumption (including changing the choice architecture and nudging), and informing, educating, promoting or empowering through community initiatives, labelling and other means^(^ ^30^ ^)^.

## Conclusion

The present study is the first to compare how well diets with varying levels of carbon footprint adhere to all nutrient recommendations in the NNR. Although differences were small, we found that participants with the smallest carbon footprint, despite a higher intake of added sugar, adhered to a larger number of nutrient recommendations than those with the largest footprint. Thus, we can conclude that persons in Sweden with a diet low in GHGE have a slightly more nutritious diet overall compared with those whose diet is associated with high emissions.

## Supplementary material

For supplementary material accompanying this paper visit https://doi.org/10.1017/S1368980017002300. click here to view supplementary material
