# Supplementary material for: Adherence to dietary recommendations for Swedish adults across categories of greenhouse gas emissions from food
Source: Public Health Nutr. 2017 Sep 7;20(18):3381–93. doi: 10.1017/S1368980017002300 (PMC10261478; doi:10.1017/S1368980017002300)
Supplement: Supplementary file 1 [file S1368980017002300sup001.docx]

| **Page** | **Content** | **Description** |
| --- | --- | --- |
| **2-7** | **Table S1 + References** | Life cycle assessment (LCA) data of greenhouse gas emissions (GHGE) expressed as  kg carbon dioxide equivalents (CO_2_e) per kg of food products or groups. |
| **8-9** | **Table S2** | Recommended intake range and recommended thresholds for macronutrients. |
| **10-11** | **Table S3** | Estimated average requirement (AR) for micronutrients. |
| **12-13** | **Table S4** | Recommended intake (RI) for micronutrients. |
| **14** | **Fig. S1** | Visualization of estimated average requirement (AR) compared to recommended intake (RI). |
| **15-16** | **Figs S2(a) and S2(b)** | Proportion adhering to recommended intake (RI) for micronutrients, by quartiles of increasing levels of dietary GHGE, for (a) women and (b) men. |
| **17** | **Figs S3(a) and S3(b)** | Boxplots with total number of fulfilled nutrient recommendations, by quartiles of increasing levels of dietary GHGE, for (a) women and (a) men. |

| **Table S1 Life cycle assessment (LCA) data of greenhouse gas emissions (GHGE) expressed as kg carbon dioxide equivalents (CO_2_e) per kg of food products or groups.**  All original LCA studies included CO_2_e from agriculture and its inputs, and the majority also included emissions up to and including the retail phase. We adjusted original LCA data to include the same system boundaries, for example added standard emissions factors from post-farm processes (such as processing, packaging, distribution and retail). Emissions after the retail phase (transport to households, storing and cooking, as well as from waste management) or emissions related to land-use change were not included. LCA data were recalculated for weight change during food preparation, considering both hydration (i.e. cooking of rice) and dehydration (i.e. cooking of meat), and adjusted for unavoidable food losses (i.e. shell and bone) as well as for avoidable food waste both before and after food preparation. Examples of calculation of food losses and waste after the product leaves the retail as well as weight change during food preparation can be found below References for Table S1, page 7. | | |
| --- | --- | --- |
| **Food products or groups** | **Kg CO_2_e / kg food** | **Estimated from** |
| Beef, raw | 35,9 | Bryngelsson et al ^(1)^ |
| Beef, prepared (fried/boiled/smoked) | 45,9 | Bryngelsson et al ^(1)^ |
| Minced meat (mix of beef and pork 70/30), raw | 27,0 | Bryngelsson et al ^(1)^ |
| Minced meat (mix of beef and pork 70/30), prepared (fried) | 35,9 | Bryngelsson et al ^(1)^ |
| Minced meat (mix of beef and pork 50/50), raw | 21,1 | Bryngelsson et al ^(1)^ |
| Minced meat (mix of beef and pork 50/50), prepared (fried) | 28,0 | Bryngelsson et al ^(1)^ |
| Sheep meat (mutton), raw | 39,0 | Bryngelsson et al ^(1)^ |
| Sheep meat (mutton), prepared (fried) | 51,7 | Bryngelsson et al ^(1)^ |
| Game, raw | 5,2 | Cejie ^(2)^ |
| Game, prepared (fried/boiled) | 6,7 | Cejie ^(2)^ |
| Pork, raw | 6,4 | Bryngelsson et al ^(1)^ |
| Pork, prepared (fried/boiled, ham) | 9,6 | Bryngelsson et al ^(1)^ |
| Poultry, raw | 2,5 | Bryngelsson et al ^(1)^ |
| Poultry, prepared (fried/boiled) | 3,6 | Bryngelsson et al ^(1)^ |
| Whole milk | 1,5 | Bryngelsson et al ^(1)^ and Flysjö ^(3)^ |
| Semi-skimmed milk | 1,3 | Bryngelsson et al ^(1)^ and Flysjö ^(3)^ |
| Skimmed milk | 1,2 | Bryngelsson et al ^(1)^ and Flysjö ^(3)^ |
| **Table S1 continued** |  |  |
| Yoghurt | 1,6 | Bryngelsson et al ^(1)^ and Flysjö ^(3)^ |
| Yoghurt low fat | 1,4 | Bryngelsson et al ^(1)^ and Flysjö ^(3)^ |
| Crème fraiche | 6,3 | Bryngelsson et al ^(1)^ and Flysjö ^(3)^ |
| Crème fraiche low fat | 3,8 | Bryngelsson et al ^(1)^ and Flysjö ^(3)^ |
| Yellow cheese | 11,8 | Bryngelsson et al ^(1)^ and Flysjö ^(3)^ |
| Yellow cheese low fat | 10,8 | Bryngelsson et al ^(1)^ and Flysjö ^(3)^ |
| White cheese | 9,7 | Bryngelsson et al ^(1)^ and Flysjö ^(3)^ |
| Mould cheese | 10,0 | Bryngelsson et al ^(1)^ and Flysjö ^(3)^ |
| Cream cheese | 8,1 | Bryngelsson et al ^(1)^ and Flysjö ^(3)^ |
| Cream cheese low fat | 5,1 | Bryngelsson et al ^(1)^ and Flysjö ^(3)^ |
| Halloumi and feta cheese | 16,6 | Bryngelsson et al ^(1)^ and ^(4)^ and ^(5)^ |
| Mozzarella | 12,2 | Bryngelsson et al ^(1)^ and ^(4)^ and ^(5)^ |
| Cottage cheese | 4,2 | Bryngelsson et al ^(1)^ and Flysjö ^(3)^ |
| Cream | 6,5 | Bryngelsson et al ^(1)^ and Flysjö ^(3)^ |
| Cream low fat | 3,1 | Bryngelsson et al ^(1)^ and Flysjö ^(3)^ |
| Ice-cream | 2,6 | Nilsson et al ^(6)^ |
| Butter | 13,0 | Bryngelsson et al ^(1)^ and Flysjö ^(3)^ |
| Butter blends (Bregott) | 8,3 | Bryngelsson et al ^(1)^ and Flysjö ^(3)^ |
| Vegetable oils and margarine | 2,3 | Bryngelsson et al ^(1)^ |
| Soy drink, oat drink (coconut milk) | 0,3 | Bryngelsson et al ^(1)^ |
| Eggs | 1,3 | Bryngelsson et al ^(1)^ |
| Legumes (beans, peas and lentils), dried | 0,8 | Bryngelsson et al ^(1)^ |
| Legumes (beans, peas and lentils), soaked and boiled | 0,3 | Bryngelsson et al ^(1)^ |
| Meat substitutes (other than quorn) | 2,5 | Röös ^(7)^ and Lidell ^(8)^ |
| Quorn | 4,5 | Röös ^(7)^ |
| Fresh fruits and berries, domestic | 0,3 | Bryngelsson et al ^(1)^ |
| Fresh fruits and berries, imported (other than banana and citrus fruit) | 1,3 | Bryngelsson et al ^(1)^ |
| Fresh fruit, berries and vegetables, aviation | 18,5 | Röös ^(7)^ |
| **Table S1 continued** |  |  |
| Dried fruit | 11,7 | Bryngelsson et al ^(1)^ |
| Banana | 2,6 | Bryngelsson et al ^(1)^ |
| Citrus fruit | 1,0 | Bryngelsson et al ^(1)^ |
| Tomato | 1,0 | Bryngelsson et al ^(1)^ |
| Iceberg lettuce | 0,4 | Bryngelsson et al ^(1)^ |
| Cucumber | 1,4 | Davis ^(9)^ |
| Root vegetables, onion | 0,2 | Bryngelsson et al ^(1)^ |
| Broccoli and vegetables not included in "root vegetables, onion" | 0,9 | Bryngelsson et al ^(1)^ |
| Potatoes, raw | 0,2 | Bryngelsson et al ^(1)^ |
| Potatoes, prepared (boiled/baked/fried/pommes frites) | 0,3 | Bryngelsson et al ^(1)^ |
| Pasta, couscous, bulgur, quinoa, dried | 0,7 | Bryngelsson et al ^(1)^ |
| Pasta, couscous, bulgur, quinoa, prepared (boiled) | 0,3 | Bryngelsson et al ^(1)^ |
| Flour, grain | 0,6 | Bryngelsson et al ^(1)^ |
| Breakfast cereals | 0,7 | Bryngelsson et al ^(1)^ |
| Rice, raw | 2,1 | Bryngelsson et al ^(1)^ |
| Rice, prepared (boiled) | 0,7 | Bryngelsson et al ^(1)^ |
| Olives | 3,4 | Florén et al ^(10)^ |
| Bread and crisp bread | 0,7 | Bryngelsson et al ^(1)^ |
| Cookies and biscuits | 1,2 | Bryngelsson et al ^(1)^ and Nilsson et al ^(6)^ |
| Soft drinks, fruit syrup | 0,2 | Nilsson et al ^(6)^ |
| Juice | 0,9 | Röös ^(7)^ |
| Instant coffee powder | 8,3 | Nilsson et al ^(11)^ |
| Coffee (prepared) | 0,2 | Nilsson et al ^(11)^ |
| Tea | 0,04 | Nilsson et al ^(11)^ and Scarborough et al ^(12)^ |
| Snacks (crisps etc) | 2,3 | Nilsson et al ^(6)^ |
| Foam sweets | 4,1 | Nilsson et al ^(6)^ |
| Jelly sweets | 2,6 | Nilsson et al ^(6)^ |
|  |  |  |
| **Table S1 continued** |  |  |
| Milk chocolate | 2,9 | Nilsson et al ^(6)^ |
| Dark chocolate | 1,0 | Nilsson et al ^(6)^ |
| Sugar and syrup | 4,8 | Bryngelsson et al ^(1)^ |
| Jam | 3,8 | Röös ^(7)^ |
| Nuts and Seeds | 1,3 | Bryngelsson et al ^(1)^ |
| Wine / liqueur | 2,3 | Bryngelsson et al ^(1)^ |
| Beer | 1,1 | Bryngelsson et al ^(1)^ |
| Cider, strong | 1,1 | Bryngelsson et al ^(1)^ |
| Distilled beverage/ vodka/ rum | 3,6 | Saxe et al ^(13)^ and Amienyo ^(14)^ |
| Salmon, raw. Norwegian aquaculture. | 3,8 | Ziegler et al ^(15)^ and Winther et al ^(16)^ |
| Salmon, prepared (fried/boiled/smoked). Norwegian aquaculture. | 4,9 | Ziegler et al ^(15)^ and Winther et al ^(16)^ |
| Cod, raw. Caught in Norwegian fisheries by various fishing gears. | 3,7 | Ziegler et al ^(15)^ and Winther et al ^(16)^ |
| Cod, prepared (fried/boiled). Caught in Norwegian fisheries by various fishing gears. | 4,2 | Ziegler et al ^(15)^ and Winther et al ^(16)^ |
| Saithe, raw. Caught in Norwegian fisheries by various fishing gears. | 3,0 | Ziegler et al ^(15)^ and Winther et al ^(16)^ |
| Saithe, prepared (fried/boiled). Caught in Norwegian fisheries by various fishing gears. | 3,3 | Ziegler et al ^(15)^ and Winther et al ^(16)^ |
| Haddock, raw. Caught in Norwegian fisheries by various fishing gears. | 4,3 | Ziegler et al ^(15)^ and Winther et al ^(16)^ |
| Haddock, prepared (fried/boiled). Caught in Norwegian fisheries by various fishing gears. | 4,8 | Ziegler et al ^(15)^ and Winther et al ^(16)^ |
| Herring, raw. Caught by the Norwegian pelagic fleet. | 1,1 | Ziegler et al ^(15)^ and Winther et al ^(16)^ |
| Herring, prepared (fried/boiled/pickled). Caught by the Norwegian pelagic fleet. | 1,3 | Ziegler et al ^(15)^ and Winther et al ^(16)^ |
| Mackerel, raw. Caught by the Norwegian pelagic fleet. | 1,2 | Ziegler et al ^(15)^ and Winther et al ^(16)^ |
| Mackerel, prepared (fried). Caught by the Norwegian pelagic fleet. | 1,3 | Ziegler et al ^(15)^ and Winther et al ^(16)^ |
| Crustaceans (shrimps, lobster) without shell, boiled. Europe, bottom trawl. | 38,7 | Parker et al ^(17)^ |
| Crustaceans (shrimps, lobster), with shell, boiled. Europe, bottom trawl. | 14,7 | Parker et al ^(17)^ |
| Mussel, without shell. Norwegian aquaculture. | 1,8 | Ziegler et al ^(15)^ and Winther et al ^(16)^ |
| **Table S1 continued** |  |  |
| Tuna raw | 3,5 | Ziegler et al ^(15)^, Winther et al ^(16)^, Parker et al ^(17)^ |
| Tuna, prepared | 4,0 | Ziegler et al ^(15)^, Winther et al ^(16)^, Parker et al ^(17)^ |
| Other seafood, passive capture fisheries, raw | 0,7 | Ziegler et al ^(15)^ and Winther et al ^(16)^ |
| Other seafood, passive capture fisheries, prepared | 0,8 | Ziegler et al ^(15)^ and Winther et al ^(16)^ |
| Other seafood, active capture fisheries, raw | 2,8 | Ziegler et al ^(15)^ and Winther et al ^(16)^ |
| Other seafood, active capture fisheries, prepared | 3,1 | Ziegler et al ^(15)^ and Winther et al ^(16)^ |
| Bottled water | 0,1 | Konsumentföreningen Stockholm/SIK ^(18)^ |
| Unknown (including tap water) | 0 |  |

**References for Table S1**

1. Bryngelsson D, Wirsenius S, Hedenus F, Sonesson U. How can the EU climate targets be met? A combined analysis of technological and demand-side changes in food and agriculture. Food Policy. 2016;59:152-64.

2. Cejie J. Klimatpåverkan från vilt kött (Climate impact from game meat). 2008. <http://ekotank.blogspot.se/2008/11/klimatpverkan-frn-vilt-ktt.html> (Webcite: <http://www.webcitation.org/6RfsoOEpH>) (accessed June 2017).

3. Flysjö A. Greenhouse gas emissions in milk and dairy product chains: Improving the carbon footprint of dairy products. PhD thesis. Aarhus University, Denmark. 2012.

4. Doddridge E. The BBQ challenge <http://doddridge.me/2015/01/02/the-bbq-challenge/> (Webcite: <http://www.webcitation.org/6fAYfgpmr> ) (accessed June 2017).

5. Doddridge E. Detailed working for the different BBQ scenarios <http://nbviewer.jupyter.org/urls/dl.dropbox.com/s/2qp10d2blkkn5m9/BBQs.ipynb?dl=0> (Webcite: <http://www.webcitation.org/6fAYjgkl1> ) (accessed June 2017).

6. Nilsson K, Sund V, Florén B. The environmental impact of the consumption of sweets, crisps and soft drinks. Copenhagen: Nordic Council of Ministers; 2011. TemaNord 2011:509.

7. Röös E. Mat-klimat-listan Version 1.1 (Food-climate-list Version 1.1). In Swedish. Swedish University of Agricultural Sciences (SLU). 2014. Report 077. ISSN 1654-9406 <http://pub.epsilon.slu.se/11671/7/roos_e_141125.pdf> (Webcite: <http://www.webcitation.org/6mtTRm6Dn> (accessed June 2017).

8. Lidell A-K. Food for progress, Sweden. Personal communication. 2016.

9. Davis J, Wallman M, Sund V, Emanuelsson A, Cederberg C, Sonesson U. Emissions of greenhouse gases from production of horticultural products. Analysis of 17 products cultivated in Sweden. SIK - The Swedish Institute for Food and Biotechnology, Gothenburg, Sweden. 2011. SR 828. ISBN 978-91-7290-301-2.

10. Florén B, von Bah B, Davis J, Flysjö A, Högberg J, Lorentzon K, et al. Global warming potential for 100 ICA private label food products. Final report.; 2007. Report No.: UP-07-14423.

11. Nilsson K. Klimatpåverkan från bryggkaffe och snabbkaffe (Climate impact of filter coffee and instant coffee). In Swedish. Report UPX00221. SIK - The Swedish Institute for Food and Biotechnology, Gothenburg, Sweden. 2010.

12. Scarborough P, Appleby P, Mizdrak A, Briggs A, Travis R, Bradbury K, et al. Dietary greenhouse gas emissions of meat- eaters, fish- eaters, vegetarians and vegans in the UK. Climatic Change. 2014;125(2):179-92.

13. Saxe H. LCA-based comparison of the climate footprint of beer vs. wine & spirits. Fødevareøkonomisk Institut, Københavns Universitet. (Report; No. 207). 2010.

14. Amienyo D. Life Cycle Sustainability Assessment in the UK Beverage Sector. A thesis submitted to the University of Manchester for the degree of Doctor of Philosophy in the School of Chemical Engineering and Analytical Science. 2012.

15. Ziegler F, Winther U, Hognes E, Emanuelsson A, V VS, H HE. The carbon footprint of Norwegian seafood products on the global seafood market. Journal of Industrial Ecology. 2013;17(1):103-16.

16. Winther U, Ziegler F, Hognes E, Emanuelsson A, Sund V, Ellingsen H. Carbon footprint and energy use of Norwegian seafood products. SINTEF Fisheries and Aquaculture, Report SFH80 A. 2009;96068.

17. Parker RWR, Tyedmers PH. Fuel consumption of global fishing fleets: current understanding and knowledge gaps. Fish and Fisheries. 2015;16(4):684-96.

18. Konsumentföreningen Stockholm (Stockholm Consumers Association)/SIK – Institutet för Livsmedel och Bioteknik. Vatten på flaska onödig lyx. Totala koldioxidutsläpp från konsumtionen av buteljerat vatten i Sverige. In Swedish. (Bottled water unnecessary luxury. Total carbon dioxide emissions from the consumption of bottled water in Sweden.) 2007.

| **Example of calculation of food losses and waste after the product leaves the retail, for Table S1**  Adjustment for food losses and waste after the retail for banana:  1,33 kg CO_2_e per kg banana with peel/skin (until the product leaves the retail).  37% unavoidable losses (banana peel/skin). 1,33 / (1-0,37) = 2,11.  2,11 kg CO_2_e per kg banana without peel/skin (i.e. including unavoidable losses).  18% avoidable waste. 2,11 / (1-0,18) = 2,57.  2,57 kg CO_2_e per kg banana including unavoidable losses and avoidable waste. | **Example of calculation of weight change during food preparation, for Table S1**  Adjustment for weight change during food preparation for rice:  1,8 kg CO_2_e per kg dry (unprepared) rice, including food losses and waste.  Dry weight in dry (unprepared) rice: 89 g per 100 g.  Dry weight in boiled (prepared) rice: 31 g per 100 g.  Weight change during food preparation for boiled (prepared) rice: 89/31=2,87.  1,8 / 2,87 = 0,627 kg CO_2_e per kg boiled (prepared) rice including food losses and waste before food preparation and weight change during food preparation  (but excluding food waste after food preparation). |
| --- | --- |

**Table S2** *Recommended intake range* and *recommended thresholds* of macronutrients, for women and men with different age intervals,

from the Nordic Nutrition Recommendations 2012 - Integrating nutrition and physical activity.

| **Nutrients** | **Recommended intake range and thresholds^*^** | |
| --- | --- | --- |
|  | **Women** | **Men** |
| Protein (E%)^†,‡^ | 10-20/15-20 | 10-20/15-20 |
| Carbohydrates (E%)^†^ | 45-60 | 45-60 |
| Dietary fibre (g/1000 kJ) | 3 | 3 |
| Added sugar (E%)^†,§^ | <10 | <10 |
| Total fat (E%)^†^ | 25-40 | 25-40 |
| SFA (E%)^†^ | <10 | <10 |
| MUFA (E%)^†^ | 10-20 | 10-20 |
| PUFA (E%)^†,^ ^¶^ | 5-10 | 5-10 |
| MUFA and PUFA^ǁ^ | 2/3 of total FA | 2/3 of total FA |
| *n*-3 FA (E%)^†,**^ | 1 | 1 |
| Linoleic acid (LA) and |  |  |
| α-Linolenic acid (ALA) (E%)^†,††^ | 3 | 3 |

Abbreviations: E%, energy percent; FA, fatty acids.

^*^ Recommended intake range for protein, carbohydrates, total fat, MUFA and PUFA. Lower thresholds for dietary fibre, “MUFA and PUFA”,

*n*-3 FA and “Linoleic acid (LA) and α-Linolenic acid (ALA)”. Upper thresholds for added sugars and SFA.

†Energy percent (E%) is calculated without including energy from alcohol. 1 gram of fat = 37 kJ, 1 gram of protein = 17 kJ, 1 gram of carbohydrate = 17 kJ.

^‡^Women and men 18-64 y: 10-20 E%, 65-80 y 15-20 E%.

^§^The amounts of sucrose and monosaccharides from natural sources were subtracted from the total amount of sucrose and monosaccharides in the diets to calculate the amount of added sugar in gram. The E% from added sugar was calculated referring to the proportion of the energy in the diet that the added sugar contributed. 1 gram of added sugar = 17 kJ.

^¶^Intake of cis-polyunsaturated fatty acids should be 5–10 E%.

^ǁ^Cis-monounsaturated and cis-polyunsaturated fatty acids should constitute at least two thirds of the total fatty acids in the diet.

^**^Total *n*-3 FA should provide at least 1 E%. To calculate total *n*-3 FA in the present study α-Linolenic acid (ALA), eicosapentaenoic acid (EPA) and docosahexaenoic acid (DHA) were summarized. *n*-3 fatty acids are also called Omega-3 FA and ω-3 FA.

^††^Linoleic (LA, *n*-6) and α-Linolenic (ALA, *n*-3) FA should contribute at least 3 E%, including at least 0.5 E% as α-Linolenic (ALA, *n*-3) FA.

**Table S3** Estimated average requirement (AR) for certain micronutrients (vitamins and minerals) for adults. AR is missing for potassium, magnesium and sodium. This table is based on table 1.8 in Nordic Nutrition Recommendations 2012 - Integrating nutrition and physical activity.

| **Nutrients** | **Average Requirement (AR)^*^** | |
| --- | --- | --- |
|  | **Women** | **Men** |
| Vitamin A RE (RE) ^†^ | 500 | 600 |
| Vitamin D (µg) | 7.5 | 7.5 |
| Vitamin E α-TE (α-TE) ^‡^ | 5 | 6 |
| Thiamin (mg) | 0.9 | 1.2 |
| Riboflavin (mg) | 1.1 | 1.4 |
| Niacin NE (NE) ^§^ | 12 | 15 |
| Vitamin B_6_ (mg) | 1.1 | 1.3 |
| Folate (µg) | 200 | 200 |
| Vitamin B_12_ (µg) | 1.4 | 1.4 |
| Vitamin C (mg) | 50 | 60 |
| Calcium (mg) | 500 | 500 |
| Phosphorus (mg) | 450 | 450 |
| Potassium (mg) ^¶^ | AR missing. LI: 1600 | AR missing. LI: 1600 |
| Magnesium (mg) ^ǁ^ | AR and LI missing. | AR and LI missing. |
| Iron (mg) ^**^ | 10/6 | 7 |
| Zinc (mg) | 5 | 6 |
| Selenium (µg) | 30 | 35 |
| Sodium (g) ^††^ | AR and LI missing. | AR and LI missing. |

Abbreviations: RE, retinol equivalents; α-TE, α-tocopherol equivalents; NE, niacin equivalent; LI, lower intake level.

^*^ The AR values are intended for use only in assessing results from dietary surveys. The definition of AR corresponds to the term ‘Estimated Average Requirement’ (EAR) used in the UK and US recommendations. The European Food Safety Authority (EFSA) uses the term ‘Average Requirement’.

^†^1 Retinol equivalent (RE) = 1 μg retinol = 12 μg β-carotene.

^‡^ 1 α-tocopherol equivalent (α-TE) = 1 mg RRR α-tocopherol.

^§^ 1 niacin equivalent (NE) = 1 mg niacin = 60 mg tryptophan.

^¶^ AR is missing for potassium. In the present study we used LI = 1600 mg. The definition of LI differs from the term ‘Lower reference nutrient intake’ (LRNI) used in the UK, which is defined as EAR minus 2 SD. The EFSA uses the term ‘Lower threshold intake’ (LTI) to define the level of intake below which almost all individuals will be unlikely to maintain ‘metabolic integrity’ according to the criterion chosen for each nutrient .

^ǁ^ AR and LI are missing for magnesium. In the present study we used RI = 280 mg for women and 350 mg for men. The definition of RI corresponds to the term ‘Recommended Intake’ used in the UK and ‘Recommended Dietary Allowance’ (RDA) used in the US. The EFSA uses the term ‘Population Reference Intake’ (PRI) to denote “the level of nutrient intake that is enough for virtually all healthy people in a group”.

^**^AR for post-menopausal women is 6 mg per day. In the present study we used the age intervals: 18-50 y: 10 mg, 51-80 y: 6 mg.

^††^ 2.4 g sodium corresponds to 6 g salt (sodium chloride, NaCl). AR and LI are missing for sodium. In the present study we used RI = ≤2.4 g for women and men. The definition of RI corresponds to the term ‘Recommended Intake’ used in the UK and ‘Recommended Dietary Allowance’ (RDA) used in the US. The EFSA uses the term ‘Population Reference Intake’ (PRI) to denote “the level of nutrient intake that is enough for virtually all healthy people in a group”.

**Table S4** Recommended intake (RI) for micronutrients, for women and men with different age intervals,

based on table 1.3 in Nordic Nutrition Recommendations 2012 - Integrating nutrition and physical activity.

| **Nutrients** | **Recommended Intake (RI)**^*^ | |
| --- | --- | --- |
|  | **Women** | **Men** |
| Vitamin A RE (RE) ^†^ | 700 | 900 |
| Vitamin D (µg) ^‡^ | 10/20 | 10/20 |
| Vitamin E α-TE (α-TE) ^§^ | 8 | 10 |
| Thiamin (mg) ^¶^ | 1.1/1.0 | 1.4/1.3/1.2 |
| Riboflavin (mg) ^ǁ^ | 1.3/1.2 | 1.6/1.5/1.4/1.3 |
| Niacin NE (NE) ^**^ | 15/14/13 | 19/18/16/15 |
| Vitamin B_6_ (mg) ^††^ | 1.2/1.3 | 1.5 |
| Folate (µg) ^‡‡^ | 400/300 | 300 |
| Vitamin B_12_ (µg) | 2.0 | 2.0 |
| Vitamin C (mg) | 75 | 75 |
| Calcium (mg)^§§^ | 900/800 | 900/800 |
| Phosphorus (mg) ^¶¶^ | 700/600 | 700/600 |
| Potassium (mg) | 3100 | 3500 |
| Magnesium (mg) | 280 | 350 |
| Iron (mg) ^ǁǁ^ | 15/9 | 9 |
| Zinc (mg) | 7 | 9 |
| Selenium (µg) | 50 | 60 |
| Sodium (g) ^***^ | ≤2.4 | ≤2.4 |

Abbreviations: RE, retinol equivalents; α-TE, α-tocopherol equivalents; NE, niacin equivalent.

^*^ RI of certain nutrients expressed as the average daily intake over time for use in planning diets for groups. The requirements are lower for almost all individuals. The definition of RI corresponds to the term ‘Recommended Intake’ used in the UK and ‘Recommended Dietary Allowance’ (RDA) used in the US . The EFSA uses the term ‘Population Reference Intake’ (PRI) to denote “the level of nutrient intake that is enough for virtually all healthy people in a group” .

†1 Retinol equivalent (RE) = 1 μg retinol = 12 μg β-carotene. (No recommended value determined for Retinol or β-carotene.)

^‡^ Women and men 18-74 y: 10 µg, 75-80 y: 20 µg.

^§^1 α-tocopherol equivalent (α-TE) = 1 mg RRR α-tocopherol.

^¶^ Women 18-60 y: 1.1 mg, 61-74 y: 1.0 mg. Men 18-30 y: 1.4 mg, 31-80 y: 1.3 mg, 61-80 y: 1.2 mg.
^ǁ^ Women 18-30 y: 1.3 mg, 31-80 y: 1.2 mg. Men 18-30 y: 1.6 mg, 31-60 y: 1.5 mg, 61-74 y: 1.4 mg, 75-80 y: 1.3 mg.

^**^ Women 18-30 y: 15 NE, 31-60 y: 14 NE, 61-80 y: 13 NE. Men 18-30 y: 19 NE, 31-60 y: 18 NE, 61-74 y: 16 NE, 75-80 y: 15 NE. 1 niacin equivalent (NE) = 1 mg niacin = 60 mg tryptophan.

^††^ Women 18-60 y: 1.2 mg, 61-80 y: 1.3 mg.

^‡‡^ Women of reproductive age are recommended to have an intake of 400 μg per day. In the present study we used the age intervals: 18-50 y: 400 μg, 51-80 y: 300 μg.

^§§^ Women and men 18-20 y: 900 mg. 21-80 y: 800 mg.

^¶¶^ Women and men 18-20 y: 700 mg, 21-80 y: 600 mg.

^ǁǁ^ Recommended intake for post-menopausal women is 9 mg per day. In the present study we used the age intervals: 18-50 y: 15 mg, 51-80 y: 9 mg.

^***^ 2.4 g sodium corresponds to 6 g salt (sodium chloride, NaCl).

**Fig. S1 Visualization of AR compared to RI.** Frequency distribution of an individual nutrient requirement.

The RI values include a safety margin accounting for variations in the physiological and dietary requirement in the population.

Therefore, most individuals in a population are likely to have a requirement lower than RI.

Fig. S1 corresponds to Figure 2.2. in the Nordic Nutrition Recommendations 2012 - Integrating nutrition and physical activity.

SD, standard deviation.

**Fig. S2(a)** Proportion of participants adhering to recommended intake (RI) of micronutrients in the Nordic Nutrition Recommendations (NNR) 2012, by quartiles of increasing levels of dietary GHGE adjusted for total energy intake, among 840 women, Riksmaten adults 2010-11 survey, Sweden. Quartile 1 is the lowest and 4 the highest GHGE group. *P* values are from χ^2^ test. GHGE, greenhouse gas emissions; CO_2_e, carbon dioxide equivalents; Vit., vitamin.

**Fig. S2(b)** Proportion of participants adhering to recommended intake (RI) of micronutrients in the Nordic Nutrition Recommendations (NNR) 2012, by quartiles of increasing levels of dietary GHGE adjusted for total energy intake, among 627 men, Riksmaten adults 2010-11 survey, Sweden. Quartile 1 is the lowest and 4 the highest GHGE group. *P* values are from χ^2^ test. GHGE, greenhouse gas emissions; CO_2_e, carbon dioxide equivalents; Vit., vitamin.

**Fig. S3** Boxplots with total number of fulfilled recommendations in the Nordic Nutrition Recommendations 2012, by quartiles of increasing levels of GHGE adjusted for total energy intake, among (a) 840 women and (b) 627 men, Riksmaten adults 2010-11 survey, Sweden. Quartile 1 is the lowest and 4 the highest GHGE group. GHGE, greenhouse gas emissions; CO_2_e, carbon dioxide equivalents.
